# Efficacy of RADA16-Based Self-Assembling Peptides on Wound Healing: A Meta-Analysis of Preclinical Animal Studies

**DOI:** 10.3390/ph18040526

**Published:** 2025-04-03

**Authors:** Jiaju Lu, Liuting Chen, Zeyue Sun, Zhimou Yang

**Affiliations:** 1 Key Laboratory of Bioactive Materials, Ministry of Education, State Key Laboratory of Medicinal Chemical Biology, College of Life Sciences, Nankai University, Tianjin 300071, China; yangzm@nankai.edu.cn; 2 School of Materials Science and Engineering, Zhejiang Sci-Tech University, Hangzhou 310018, China; 15868177627@163.com (L.C.); interstellar51@163.com (Z.S.)

**Keywords:** wound healing, RADA16, self-assembling peptides, meta-analysis

## Abstract

**Objectives**: This analysis aims to provide evidence supporting the feasibility of clinical application of self-assembling peptides for skin wound healing. **Methods**: This review was conducted following the Preferred Reporting Items for Systematic Reviews and Meta-Analyses (PRISMA) guidelines. PubMed, Web of Science, and Cochrane Library were searched (up to June 17, 2024). The primary outcome, wound closure rate at 7 and 14 days post-injury, was pooled using a random-effects meta-analysis. The risk of bias (ROB) assessment and meta-analysis were performed using the Systematic Review Centre for Laboratory animal Experimentation (SYRCLE)’s ROB tool for animal studies and RevMan software. **Results**: A total of 502 unique records were identified from our search, with 12 experimental animal studies meeting the prespecified inclusion criteria (n = 272 animals). The RADA16 interventions promoted wound closure rate compared to controls (saline or no treatment group) in both diabetic and non-diabetic animal models (Mean Difference (MD) = 11.25, 95% Confidence Interval (CI): 5.73 to 16.78, *p* < 0.0001; MD = 9.48, 95% CI: 4.75 to 14.22, *p* < 0.0001 at 7 and 14 days post-injury, respectively). Healing was further enhanced using RADA16-based functional self-assembling peptides compared to RADA16 group in both diabetic and non-diabetic animal models (MD = 27.25, 95% CI: 22.68 to 31.83, *p* < 0.00001; MD = 29.11, 95% CI: 24.30 to 33.91, *p* < 0.00001 at 7 and 14 days after injury, respectively). The ROB was uncertain for most studies due to insufficient reporting. **Conclusions**: RADA16-based self-assembling peptides, particularly those modified with functional peptide motifs, represent a promising treatment for non-diabetic and diabetic wounds in pre-clinical studies, and translation to the clinical domain appears warranted.

## 1. Introduction

Skin, as the first-line barrier of the body, plays a key role in defense against pathogenic microbes and toxic substances from external environment [1]. Cutaneous wounds can be caused by various external factors, including accidents, surgery, burn, chemical reagents, and diseases. Wound healing is a complex pathophysiological process involving a series of phases of hemostasis, inflammation, proliferation, and remodeling [2]. Several pathological conditions, such as infection, diabetes, and excessive skin pressure, can disrupt the wound-healing process and lead to a loss of the skin’s self-healing capacity [3]. The prolonged healing process imposes increased pain, decreased quality of life, and disability on patients. However, there is currently no standardized wound care that guarantees improved healing outcomes [4]. Therefore, developing effective therapeutics for skin wound healing is of great significance.

Currently, various biomaterial-based hydrogel dressings have been developed for treating acute and chronic wounds, which are based on peptides, chitosan, hyaluronic acid, and collagen. Of these, self-assembling peptide-based materials have received extensive attention [5]. First, peptides and amino acids are inherently biocompatible and biodegradable, which can be cleared by the host. They are not derived from animal sources, thereby minimizing the risk of carrying biological pathogens or contaminants commonly associated with animal-derived biomaterials [6]. Second, self-assembling peptides can be easily modified to introduce specific functionalities, such as targeted drug delivery or cell adhesion sites, offering a high degree of customization [7]. Third, the ability of self-assembling peptides to spontaneously form well-ordered, nanostructured networks allows for the creation of materials with unique properties (e.g., enhanced mechanical strength or responsiveness to environmental stimuli), which is often difficult to achieve with conventional methods [8]. Finally, unlike many synthetic biomaterials that require complex and costly fabrication methods, peptide-based materials can be synthesized relatively easily and cost-effectively on a large scale, making them an attractive option for industrial and clinical applications. Hence, self-assembling peptide-based materials are suitable candidates for wound healing.

**Figure 1:**
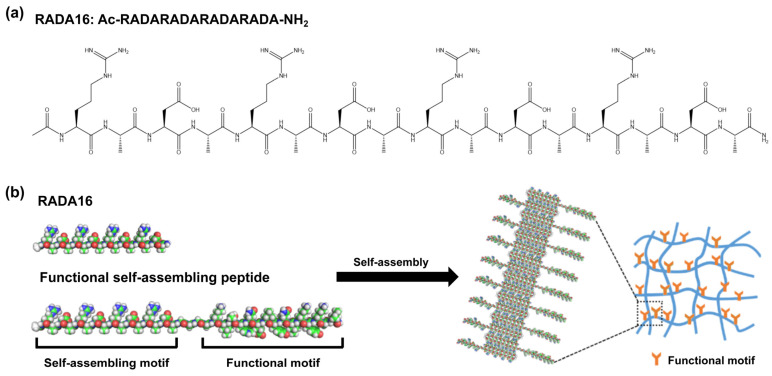
Characterizations of RADA16-based self-assembling peptides. (**a**) The molecular structural formula of RADA16; (**b**) Model representing a β-sheet structure of a functional self-assembling peptide nanofiber with active motif. The functional peptide motif extends out from the nanofiber scaffold backbone.

RADA16 (RADARADARADARADA) is a representative type of self-assembling peptide, composed of 16 alternating positively charged arginine (R), hydrophobic alanine (A), and negatively charged aspartic acid (D) residues [9]. The periodically repeating ionic peptides have charged residues on one side and hydrophobic side chains on the other, leading to the formation of extremely stable β-sheet structure [10,11] (Figure 1). This structure allows RADA16 to spontaneously self-assemble into interwoven nanofibers and eventually form a three-dimensional (3D) hydrogel in the presence of neutral pH solutions or under physiological conditions. The RADA16 hydrogel has an extremely high water content (>99% *w*/*v* water) with pore sizes ranging from 5 to 200 nm, which provides a biomimetic extracellular matrix (ECM) structure [12]. Furthermore, these hydrogels have the capability to deliver signaling molecules and functional proteins that promote cell growth and migration [13]. RADA16 has the potential for molecular-level programmability, and can be easily modified at the single amino acid level [14]. RADA16-based functional self-assembling peptide can be introduced by directly extending the RADA16 sequence with various bioactive motifs (e.g., cell adhesion [15], angiogenesis [16], and skin regeneration [17]), giving the hydrogel scaffold the ability to influence cellular behavior. Several studies showed that RADA16-based functional self-assembling peptides are considered to exert a significantly favorable influence on skin wound healing [5,18]. However, there is currently no comprehensive quantitative research on this topic.

Here, we analyzed the preclinical animal studies, including the study design, animal model, wound size, and implantation period, on the efficacy of RADA16-based self-assembling peptides in skin wound healing. The objective of this study was to identify the accelerated wound contraction potentials of RADA16-based self-assembling peptides on wound repair through a meta-analysis and systematic review. We expect that the meta-analysis of this evidence from preclinical animal research will promote further research and provide insights into the likelihood of clinical translation.

## 2. Results

### 2.1. Search Results

**Figure 2:**
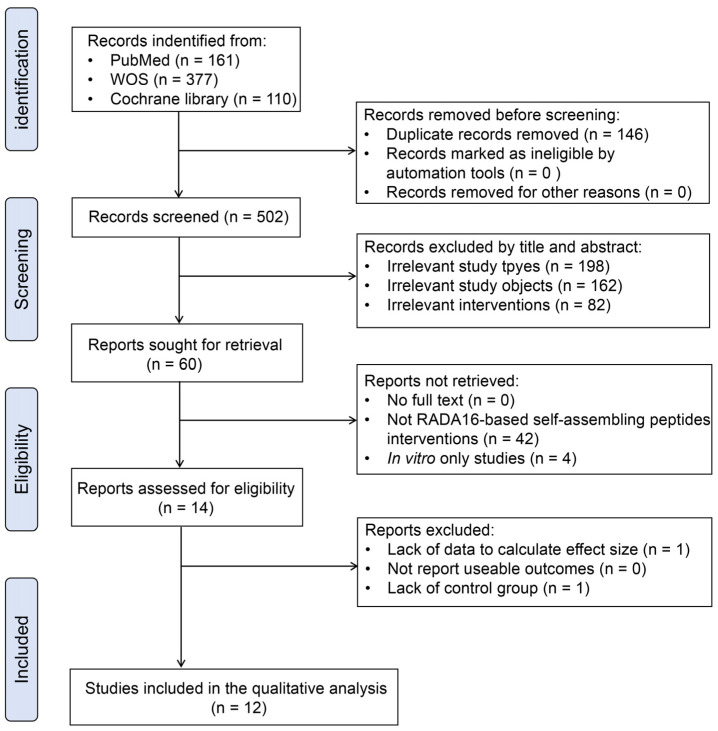
PRISMA flow chart summarizing study screening and selection procedure.

We electronically searched the articles published until June 2024. A total of 502 unique records were retrieved. After eliminating duplicates and screening the titles and abstracts for potential relevance, 442 studies were excluded as they were reviews, case series or case reports, letters, conference proceedings, or articles unrelated to the research topic. The remaining 60 studies were read in full to determine their eligibility. As a result, 48 studies were excluded for various reasons: 42 studies did not use RADA16-based self-assembling peptides as their interventions, 4 studies reported only in vitro findings, and 2 studies were excluded due to lack of data or control group. In total, we included 12 studies that addressed the therapeutic use of RADA16-based self-assembling peptides in wound healing for qualitative analysis. The detailed selection process for included studies is illustrated in Figure 2.

### 2.2. Study Characteristics

The 12 included studies were published between 2009 and 17 June 2024. Their general characteristics, including study details, basic parameters of animal models, and outcome measures, are presented in Table 1. Approximately 66.7% (n = 8) were published in 2020 or later, reflecting a surge in interest in self-assembling peptides to promote wound healing and skin regeneration. Six studies were carried out in China [19,20,21,22,23,24], two in the United States [25,26], two in the Poland [27,28], one in the Korea [29], and one in India [30]. The included studies used rats (n = 7), mice (n = 4), and swine (n = 1). Four studies were performed on animals with diabetes, and one of these used both diabetic and non-diabetic animal models [24]. In addition, eight studies were performed in animals without diabetes. Different animal models were used to perform skin wounds, including full-thickness excision wound (n = 8), burns (n = 2), Staphylococcus aureus-infected wounds (n = 1), and porcine skin puncture injury (n = 1). Wound size ranged in diameter from 6 to 30 mm, and the timing of outcome assessments ranged from 1 to 90 days post-surgery for all outcomes. Seven of the included studies used functional self-assembling peptides, with their amino acid compositions and description shown in Table 2. These functional peptide motifs are derived from the active center of the functional protein or growth factors, which have various bioactivities such as antibacterial property and the stimulation of cell migration and proliferation. They vary in length, ranging from short motifs with 3 amino acids (e.g., RGD) to longer motifs fused with up to 15 amino acids (e.g., KLT) [23]. Most active motifs were modified at the C-terminal of RADA16 (n = 6), with only one study introducing the functional peptide motifs to the N-terminal of RADA16 [23].

### 2.3. ROB Assessment of the Included Articles

**Figure 3:**
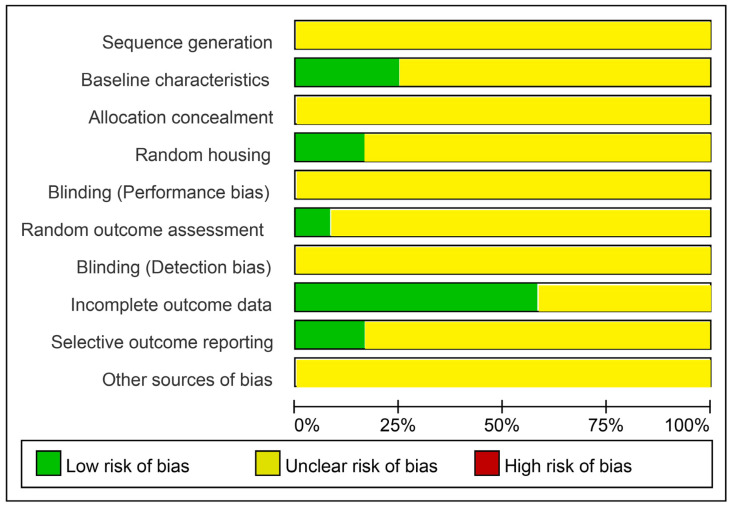
Summary of ROB of the included animal studies.

The methodological quality of the included studies was assessed by SYRCLE’s ROB tool, and the summary of ROB is presented in Figure 3. Overall, most domains were assessed as having unclear ROB in the majority of studies. All studies had an unclear risk of bias for sequence generation, as none of them provided detailed randomization methods. Three studies provided sufficient information about baseline characteristics of animal models and timing of disease induction [24]. None of the studies clarified whether allocation was concealed or whether blinding of outcome assessors was implemented consistently. Two studies reported a random approach when housing the animals within the facility [20,29]. About 58.3% of the studies showed a low ROB regarding incomplete outcome data. In addition, scatter plot asymmetry and statistical tests for the publication bias were not performed due to the limited number of studies included in our quantitative analyses. No studies were disqualified due to poor quality.

### 2.4. Outcomes of Meta-Analysis

Table S1 shows the in vivo analysis and primary quantitative results of the selected studies. Eight studies (66.7%) investigated wound closure rate, and three studies investigated collagen density. Other common evaluations by the authors included re-epithelialization (thickness of epidermis), scar assessment (α-SMA+ cell), and angiogenesis (CD31).

**Figure 4:**
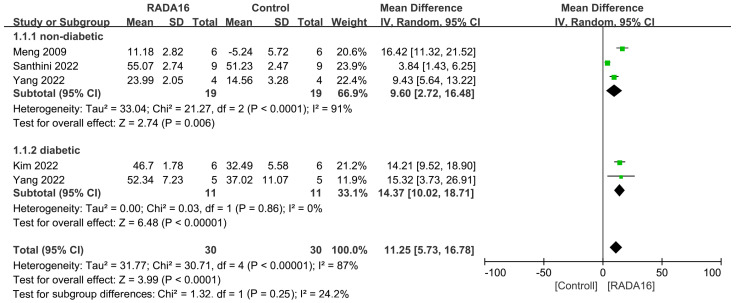
Forest plot of mean difference in wound closure rate at 7 days after injury following RADA16 interventions in diabetic or non-diabetic skin wound models in comparison to controls (saline or without treatment) [20,24,26,30].

Four studies involving 70 animals evaluated the wound closure rate between the group using RADA16 and the control group (saline or no treatment) at 7 days after injury. An overall significant enhancement of wound closure rate was observed for wounds treated with RADA16 (MD = 11.25, 95% CI: 5.73 to 16.78, *p* < 0.0001) compared to controls (Figure 4). The combined results showed significant heterogeneity (I^2^ = 87%). Subgroup meta-analyses in both non-diabetic and diabetic animals show that RADA16 was significantly more effective than the control group in accelerating wound closure (MD = 9.60, 95% CI: 2.72 to 16.48, *p* = 0.006; MD = 14.37, 95% CI: 10.02 to 18.71, *p* < 0.00001 in non-diabetic and diabetic models, respectively). High heterogeneity was observed in the non-diabetic subgroup (I^2^ = 91%).

**Figure 5:**
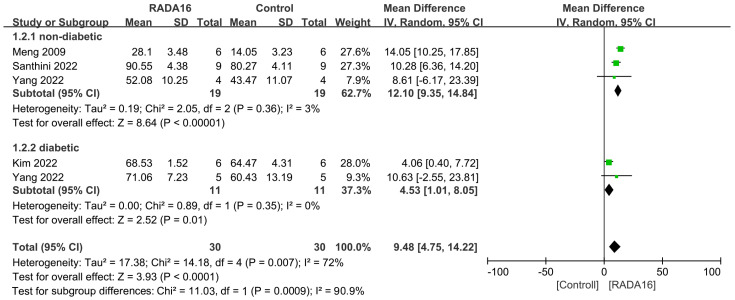
Forest plot of mean difference in wound closure rate at 14 days after injury following RADA16 interventions in diabetic or non-diabetic skin wound models in comparison to controls (saline or without treatment) [20,24,26,30].

Figure 5 presents the results of a meta-analysis on wound closure rate between RADA16 and control groups at 14 days post-injury. Pooled results suggested that RADA16 treatment was associated with a significant increased wound closure rate compared to controls (MD = 9.48, 95%CI: 4.75 to 14.22, *p* < 0.0001). The combined results showed significant heterogeneity (I^2^ = 72%). Subgroup meta-analyses indicated that RADA16 was significantly more effective than the control group in accelerating wound closure (MD = 12.10, 95% CI: 9.35 to 14.84, *p* < 0.00001; MD = 4.53, 95% CI: 1.01 to 8.05, *p* = 0.01 in non-diabetic and diabetic models, respectively). However, low heterogeneity was observed in the non-diabetic subgroup (I^2^ = 3%), which contrasts with the result at 7 days.

In Figure 4 and Figure 5, a significant heterogeneity of the combined results is observed. Of these included studies, Yang et al. [24] utilized mice, whereas other studies employed rats. Given that the type of animal species may impact wound closure rates, we excluded Yang et al.’s data and reanalyzed the efficacy of RADA16 versus the control group within the same animal species (rats) [24]. Figure S1 presents the results of a meta-analysis on wound closure rate between RADA16 and control groups at 7 days post-injury. Pooled results suggested that RADA16 treatment was associated with a significant increased wound closure rate compared to controls (MD = 11.27, 95% CI: 2.58 to 19.97, *p* = 0.01). The combined results showed significant heterogeneity (I^2^ = 93%). As shown in Figure S2, an overall significant enhancement of wound closure rate was observed for wounds treated with RADA16 (MD = 9.44, 95%CI: 3.62 to 15.26, *p* = 0.001) compared to controls at 14 days after injury. The combined results also showed significant heterogeneity (I^2^ = 86%).

**Figure 6:**
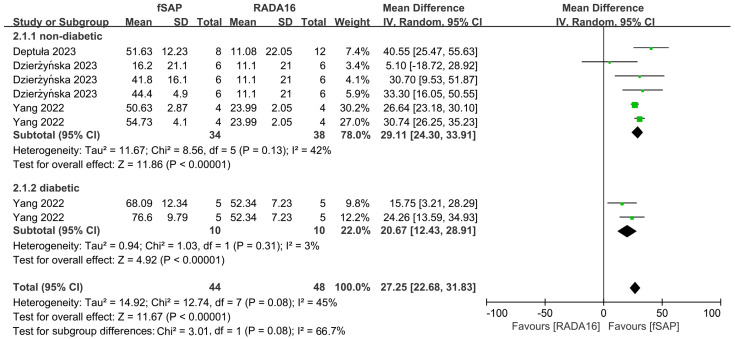
Forest plot of mean difference in wound closure rate at 7 days after injury following functional self-assembling peptide (fSAP) interventions in diabetic or non-diabetic skin wound models in comparison to RADA16 [24,27,28].

To clarify the effectiveness of RADA16-based functional self-assembling peptides on wound healing in animal models, we evaluated the effects of these peptides in three studies comprising eight independent experiment animal studies. The results (Figure 6) suggested that the wound closure rate at 7 days post-injury was significantly increased in the functional self-assembling peptides intervention group compared to unmodified RADA16 groups in both non-diabetic and diabetic animal models (MD = 27.25, 95% CI: 22.68 to 31.83, *p* < 0.00001). Moderate heterogeneity was observed in the combined results (I^2^ = 45%), in contrast to the higher heterogeneity observed in the overall analysis between RADA16 and control groups. Similarly, subgroup meta-analyses in non-diabetic and diabetic groups demonstrated that functional self-assembling peptides therapy was significantly more effective than RADA16 in accelerating wound closure in both models (MD = 29.11, 95% CI: 24.30 to 33.91, *p* < 0.00001; MD = 20.67, 95% CI: 12.43 to 28.91, *p* < 0.00001 in non-diabetic and diabetic models, respectively). Heterogeneity was moderate or low in subgroups regardless of the disease model (I^2^ = 42%, and 3% in non-diabetic and diabetic models, respectively).

**Figure 7:**
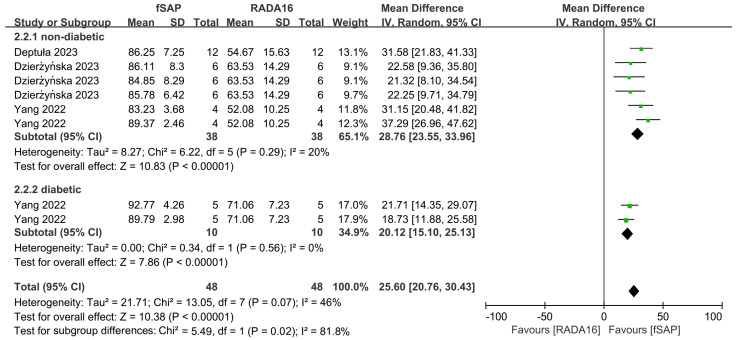
Forest plot of mean difference in wound closure rate at 14 days after injury following functional self-assembling peptide (fSAP) interventions in diabetic or non-diabetic skin wound models in comparison to RADA16 [24,27,28].

Figure 7 shows that functional self-assembling peptides significantly increased wound closure rate compared with the RADA16 group at 14 days after injury (MD = 25.60, 95% CI: 20.76 to 30.43, *p* < 0.00001). There was moderate heterogeneity (I^2^ = 46%) among studies. Subgroup analyses showed that functional self-assembling peptides were significantly more effective than RADA16 in accelerating wound closure in both models (MD = 28.76, 95% CI: 23.55 to 33.96, *p* < 0.00001; MD = 20.12, 95% CI: 15.10 to 25.13, *p* < 0.00001 in non-diabetic and diabetic models, respectively). Heterogeneity indices in these two subgroups were moderate (I^2^ = 20% or 46%, in the non-diabetic or diabetic groups, respectively).

## 3. Discussion

In this meta-analysis, 12 articles were included after a rigorous selection process, and the quantitative results of wound closure rates at both 7 and 14 days after injury were analyzed. To our knowledge, this is the first meta-analysis to evaluate the in vivo regeneration capability of RADA16-based self-assembling peptides in animal models of skin wounds. The primary aim was to gain insight into the potential clinical utility of RADA16-based self-assembling peptides interventions to promote skin wound healing. Based on our findings, RADA16 are effective therapeutic interventions for skin wounds in both non-diabetic and diabetic animal models, which can increase wound closure rate at 7 days after injury compared with control group. Specifically, RADA16-based functional self-assembling peptides outperformed the RADA16 group at both 7 and 14 days post-injury.

The heterogeneity in the non-diabetic subgroup between RADA16 and control groups at 7 days after injury was significant, with the I^2^ = 91% (Figure 4). To address this, we conducted a sensitivity analysis by sequentially removing each study. However, high heterogeneity is still observed. Furthermore, the heterogeneity in the non-diabetic subgroup between RADA16 and control groups at 14 days after injury was very low (I^2^ = 3%, Figure 5), which may be attributable to the limited number of studies (n = 3) in this subgroup. Additionally, various moderators, including animal species, type of wounds, follow-up period, and wound size, may explain the observed heterogeneity.

Skin wounds affect millions of patients annually, posing a significant threat to socioeconomic development around the world. The complex nature of wound healing requires the coordinated interactions of various cell types, growth factors, and physiological processes. However, traditional wound dressings (e.g., gauze, foams, and bandages) lack bioactive properties and fail to provide an optimal microenvironment for accelerated wound healing [31]. An ideal skin wound dressing should fulfill several criteria for optimal function [32]: (1) non-toxicity, (2) maintenance of a moist environment, (3) easy removability post-healing, (4) adequate gaseous exchange between the wounded tissue and the external environment, (5) absorption of wound exudates, and (6) biological activity, such as stimulation of angiogenesis, protection against bacterial infection, enhancement of epidermal migration, and acceleration of the wound-healing process. The RADA16 peptides and their degradation products are non-toxic, and can be enzymatically degraded and resorbed by the body without adverse effects [33]. They can be readily modified with functional peptide motifs to promote rapid healing of wounds. In addition, RADA16 peptides self-assemble into nanofibrous networks that can be tailored to form hydrogel scaffolds. The hydrogels mimic natural ECM and can maintain a moist environment, which are easily removed without trauma [34]. Given these factors, RADA16-based self-assembling peptide hydrogels have emerged as promising candidates for repairing damaged skin tissue.

Wound healing typically involves several sequential and overlapping stages, with hemostasis being the first step [35]. RADA16 peptides are currently used in the clinics settings as hemostatic agents [36]. For example, PuraStat^®^ (RADA16 aqueous solution, 3D Matrix Ltd., Caluire-et-Cuire, France) is employed for hemostasis during various surgical procedures including cardiovascular [37], gastrointestinal [38], and nasopharyngeal [39] applications. RADA16 can undergo a pH-induced transformation to form a hydrogel upon contact with blood or other physiological fluids, thereby forming a complete barrier to control bleeding at the injury site [40]. Furthermore, Meng et al. reported that RADA16 can enhance the expression of basic fibroblast growth factor (bFGF) and epidermic growth factor (EGF), which stimulate the proliferation and migration of peripheral cells, and accelerate the regeneration of epidermis [20].

Functional self-assembling peptides are easily modified by introducing functional motif peptide sequence to C- or N-terminus of RADA16, which improves the function of the fusion peptide [41]. These functional motifs are often derived from the active domains of the ECM or growth factors. For example, Deptuła et al. designed a functionalized self-assembling peptide named RADA-PDGF2, composing RADA16 linked with RLIDRTNANFL motif from platelet-derived growth factor BB (PDGF-BB) [27]. Our meta-analysis demonstrates that functionalized RADA16 exhibits superior performance compared to unmodified RADA16 in wound closure rates in both non-diabetic and diabetic animal models. However, no functionalized RADA16-related products have been approved for clinical use, possibly due to the impact of introduced active motifs on the intrinsic properties of RADA16 [42]. For instance, modifications with active motifs may alter the secondary structure of RADA16, thus weakening its self-assembling capabilities [43]. The mechanical properties and rheological characteristics of hydrogels formed by functional self-assembling peptides are also changed. Therefore, further comprehensive research is needed to minimize the adverse effects of active motifs on the self-assembly properties of RADA16.

Several potential limitations of the present meta-analysis should be considered. First, only 2–3 articles were included in the statistical analysis, assessing the effects of RADA16 or functional self-assembling peptides on non-diabetic and diabetic skin wounds. Due to the limited number of included studies and small sample sizes, additional evidence is needed to confirm the efficacy of RADA16 or functional self-assembling peptides in skin wound healing. Second, most studies exhibited an unclear risk of bias, which impaired our ability to make well-informed judgments. Third, variability in peptide sequences, concentrations, and dosages of RADA16 or functional self-assembling peptides, as well as differences in outcome measurement methods, complicate direct comparisons among studies. In animal studies, diverse animal models, species, and wound sizes were employed. Fourth, several outcomes, including re-epithelialization, collagen deposition, and angiogenesis, were not analyzed due to a lack of data in the included studies. Lastly, the animal models used may not accurately represent human disease. Despite these limitations, consistent positive effects of RADA16 and functional self-assembling peptides were observed, providing relevant findings with potential clinical translational applicability.

## 4. Materials and Methods

### 4.1. Protocol and Registration

The present meta-analysis was performed in accordance with the Preferred Reporting Items for Systematic reviews and Meta-Analyses (PRISMA) guidelines [44] (see Supplemental Checklist). It was registered on the International Prospective Register of Systematic Reviews (PROSPERO) database under protocol number CRD42024555938.

### 4.2. Eligibility Criteria

We followed the population, intervention, comparison, outcome, and study design (PICOS) model [45] to identify the research question (Table S2). The present review aims to systematically retrieve and analyze experimental animal studies, investigating the efficacy of RADA16-based self-assembling peptides in skin wound healing. For this, the control treatment could be any other (placebo) treatment or unmodified self-assembling peptides (RADA16) control. Thus, only studies that complied with the following criteria are included: (i) RADA16-based self-assembling peptides as interventions; (ii) animal studies of wound healing as study objects and (iii) controlled studies.

### 4.3. Search Strategy

This research intended to conduct a systematic review to identify animal studies addressing the use of RADA16-based self-assembling peptides for skin wound repair. A literature search was independently conducted by two investigators (J.L. and L.C.) using the following databases: PubMed, Web of Science, and Cochrane Library (Cochrane Central Register of Controlled Trials and Cochrane Database of Meta-Analysis, Cochrane Review, Trials). The search query was adjusted as needed to function with each respective database. The retrieval method combined medical subject heading (MeSH) and free words, and the keywords were defined as follows: “self-assembling peptide, RADA16, wound healing, skin repair, skin regeneration, skin rejuvenation, animal, in vivo”. The search strategy for the PubMed database is presented in Table S3. The languages and initial time periods of the searches were not limited, with a deadline of 17 June 2024. In addition, the references of included studies were inspected for relevant studies. All references were organized and managed using reference management software Citavi 6.18.0.1 (Swiss Academic Software GmbH, Wädenswil, Schweiz).

### 4.4. Study Selection

Two trained reviewers (J.L. and L.C.) independently screened retrieved reports for eligibility in accordance with the inclusion/exclusion criteria, first by title and then by abstract screening after removing duplicates. Later, by reading the full text of the article, only the articles that investigated the effect of RADA16-based self-assembling peptides on skin function and structural repair were included. In case of disagreement in article selection, a third author (Z.S.) would decide. Some articles were excluded for reasons such as being reviews, case series or case reports, letters not representing experimental studies, or primary outcomes unrelated to skin wound healing or irrelevant outcomes.

### 4.5. Assessment of Risk of Bias (ROB)

Based on the Systematic Review Centre for Laboratory Animal Experimentation (SYRCLE)’s ROB tool for animal intervention studies [46], two trained review authors (J.L. and L.C.) independently evaluated and cross-checked the inherent risk of bias in the included studies. Ten questions in this ROB tool were considered for each included study, including selection bias, performance bias, detection bias, attrition bias, report bias, and other biases. The answer to these assessment questions should be either ‘‘yes” that indicated low ROB, or ‘‘no” that indicated high ROB. Furthermore, a response with ‘‘unclear” was assigned for unclear items. Any disagreements were resolved through discussion or decided by a third reviewer (Z.S.).

### 4.6. Data Extraction and Synthesis

Data for the meta-analysis were extracted from figures using GetData Graph Digitizer 2.26, tables, and the test from the articles, and the change in mean and standard deviation between the baseline and final values were used for the meta-analysis. The following data were independently extracted by two authors (J.L. and L.C.) using a standardized data extraction sheet: (a) Study characteristics (first author, year of publication, and country); (b) Basic parameters of the included studies: animal species, age, weight, sample size, the type of wound, skin defect model, defect size, and follow-up durations of the experimental animals; (c) Outcome measures for skin defect repair: wound closure rate. Any disagreements were settled by consensus (Z.S.).

### 4.7. Statistical Analysis

Statistical analyses were performed using Review Manager (Revman, version 5.4.1) software provided by the Cochrane Collaboration (www.cochrane.org). Data were recorded as mean and standard deviation (SD), and effect size with 95% confidence interval (95% CI) was calculated. Furthermore, statistical heterogeneity was assessed using a chi-square test and the I^2^ statistic. Random-effects meta-analyses were performed because of the exploratory nature in animal studies. To test the robustness of the results, sensitivity in the included studies was performed by excluding studies one by one. The subgroup analyses were performed between animals with and without diabetes in animal studies. Finally, *p* < 0.05 was considered significant for all analyses.

## 5. Conclusions

In conclusion, the results of this meta-analysis of preclinical studies demonstrate that RADA16-based functional self-assembling peptides can effectively improve skin wound healing in both non-diabetic and diabetic animal models, as evidenced by increased the wound closure rate at 7 and 14 days post-injury. Future research should build on the insights from this meta-analysis to design robust preclinical studies that address potential sources of bias and standardize outcome measures, thereby facilitating the clinical translation of functional self-assembling peptides.

## Figures and Tables

**Table 1:** Summary of characteristics of the included studies.

Study/Year/ Ref./Country	Animal Type	Strain	Sex	Age	Weight	Sample Size	Wound Model	Wound Size	Intervention	Control Group	Follow-up Period	In Vivo Findings
Deptuła, 2023 [27] Poland	Mouse	BALB/c	Female	8-week-old	--	N_e_ = 8; N_c_ = 12	Full-thickness dorsal skin wound	6.0 mm	RADA-PDGF2	RADA16	At 2, 4, 7, 9, 11, 14, 18, and 21 days	RADA-PDGF2 accelerated wound closure in the mouse model compared to RADA16
Dzierżyńska 2023 [28] Poland	Mouse	BALB/c	Female	8–10-week-old	--	N_e_ = 18; N_c_ = 6	Full-thickness dorsal skin wound	6.0 mm	RADA-IM, RADA-GHK, and RADA-KGHK	RADA16	At 2, 4, 7, 9, 11, 14, and 18 days	RADA-GHK and RADA-KGHK peptide hybrids improved skin wound healing; RADA-IM stimulates the growth of hair follicles
Feng, 2022 [19] China	Rat	Sprague Dawley	Male	--	200–250 g	N_e_ = 12; N_c_ = 4	Full-thickness dorsal skin wound	10 mm	PNI/RA-Amps3 group, PNI/RA-Amps3/E group, and commercial dressing group	Normal saline	At 1, 3, 5, 7, 9, 11, and 13 days	PNI/RA-Amps/E hydrogel accelerated wound healing significantly compared with commercial dressing
Hsu, 2015 [25] USA	Swine	Yorkshire	Male	--	42 kg	N_e_ = 7; N_c_ = 6	Porcine skin injury	8 mm	(RADA16/HA)_200_ group	Wounds without treatment	2 min time period	(RADA16/HA)_200_ group accelerated hemostasis in porcine skin wounds as compared to plain gauze.
Kim, 2018 [29] Korea	Rat	Sprague–Dawley	--	--	200–250 g	At 7 and 21 days: N_e_ = 15; N_c_ = 5 At 14 days: N_e_ = 9; N_c_ = 3	Full-thickness skin wounds, with STZ-induced diabetes	10 mm	RADA, RADA and soluble substance P, RADA and substance P conjugated RADA	PBS	At 7, 14, and 21 days	RADA with substance P promoted wound healing to enhance skin regeneration without cell transplantation in a diabetic model
Kim, 2022 [26] USA	Rat	Sprague–Dawley	--	--	--	N_e_ = 24; N_c_ = 6	Full-thickness skin wounds, with STZ-induced diabetes	8 mm	RADA16, Slan low, Slan high, and K2 group	PBS	At 3, 7, 10, 14, 17, 21, 24, and 28 days	SLan groups showed similar wound contraction as control groups (RADA16, PBS, and K2), but increased deposition of new mature blood vessels.
Meng, 2009 [20] China	Rat	Sprague–Dawley	Female	--	250–290 g	N_e_ = 24; N_c_ = 6	Deep second degree burn wound model	3.0 cm	RADA16, Chitosan, PDLA, and Collagen	Saline	At 4, 7, 10, 14, 18, and 21 days	RADA16 dressings reduced the edema of the burn wound, speed up the beginning and disappearance of eschar and accelerate wound contraction
Santhini, 2022 [30] India	Rat	Rattus norvegicus	--	--	250–300 g	N_e_ = 9; N_c_ = 9	Excision wounds infected with S. aureus (1 × 105 CFU/mL)	1.5 cm (width) × 0.2 cm (depth)	SAP nanohydrogel	Wounds without treatment	At 7, 14, and 21 days	SAP-GF nano hydrogel completely healed the infected wounds compared to the control
Wang, 2020 [21] China	Rat	Sprague–Dawley	Female	--	--	--	Burn wound model	--	RADA16	NaCl	At 5, 10, 15, 30, 40, 50, and 90 days	RADA16 greatly promoted the healing of burn wounds
Wang, 2022 [22] China	Rat	Sprague–Dawley	--	--	200 g	N_e_ = 3; N_c_ = 3	Full-thickness skin wounds model infected with 1×10^10^ E. coli and 1 × 10^10^ S. aureus	8 mm	BASP hydrogel	Wounds without treatment	At 2, 6, 10, and 14 days	BSAP hydrogel had remarkably antibacterial ability and accelerate the wound-healing
Xue, 2022 [23] China	Mouse	NOD/SCID	--	6–8 weeks old	20–25 g	N_e_ = 20; N_c_ = 5	Full-thickness diabetic skin wounds	8 mm	hUC-MSCs, hUC-MSCsp, hUC-MSCs +hydrogel, and hUC-MSCsp+hydrogel groups	PBS	At 3, 7, 10, 14, and 21 days	Nanopeptide hydrogels loaded with hUC-MSCsp accelerated diabetic skin wound healing by inhibiting inflammation and promoting angiogenesis compared with conventional stem cell transplantation.
Yang, 2022 [24] China	Model 1 and 2: mouse	Model 1 and 2: Sprague–Dawley	Model 1 and 2: male	Model 1: 7-week-old; Model 2: Eight to ten-week-old	Not mentioned	Model 1: N_e_ = 12; N_c_ = 4 Model 2: N_e_ = 15; N_c_ = 5	Model 1: full-thickness dorsal wounds; Model 2: full-thickness skin wounds, with STZ-induced diabetes	Model 1 and 2:10 mm	Model 1 and 2: RADA16, 5% R-GHK-Cu, and 10%R-GHK-Cu	Model 1 and 2: PBS	At 3, 6, 9, 12, and 15 days	The functionalized nanofiber scaffolds significantly accelerated wound closure, collagen deposition, and tissue remodeling both in healthy and diabetic mice

N_e_, number of experiments; N_c_, number of controls; ‘—’ means not mentioned.

**Table 2:** Summary characteristics of functional self-assembling peptides.

Study/Year/Source	Name	Peptide Sequence	Description
Deptuła, 2023 [27]	RADA-PDGF2	Ac-(RADA)_4_-GGG-AAPV-GGG-RLIDRTNANFL-NH_2_	From platelet-derived growth factor BB (PDGF-BB)
Dzierżyńska, 2023 [28]	RADA-IM	Ac-(RADA)_4_-GGG-AAPV-GGG-RDKVYR-NH_2_	Imunofan (IM) that stimulates migration of keratinocytes
	RADA-GHK	Ac-(RADA)_4_-GGG-AAPV-GG-GHK-NH_2_	A primary regulatory factors of metalloproteinases and their inhibitors
	RADA-KGHK	Ac-(RADA)_4_-GGG-AAPV-GGG-KGHK-NH_2_	A primary regulatory factors of metalloproteinases and their inhibitors
Feng, 2022 [19]	RA-Amps3	Ac-RADARADARADARADA-Acp-RRWRVIVKW	An antibacterial peptide
Kim, 2018 [29]	RADA-SP	Ac-RARADADARARADADA-GG-RPKPQQFFGLM-NH_2_	Substance P secreted from the peripheral terminals of sensory nerve fibers as a neurotransmitter or hormone
Wang, 2022 [22]	ERC	RADARADARADARADA-GGQQLK	Enzyme-reaction chain
	CBC	RADARADARADARADA-GSVLGYIQIR	Calcium binding chain
Xue, 2022 [23]	KLT	GGGKLTWQELYQLKYKGI-RADARADARADARADA-NH_2_	From a VEGF mimetic fragment that activates VEGF receptors and VEGF-related cellular signaling pathways, to activate endothelial cell proliferation
	RGD	RGDRADARADARADA-NH_2_	The RGD polypeptide family is thought to have a specific recognition site for integrin receptors
Yang, 2022 [24]	R-GHK	Ac-(RADA)_4_-GG-GHK	GHK tripeptide (copper peptide) presents a strong affinity for copper ion

## Data Availability

All the data used for this publication are either presented in the main article or are available as Supplementary Information.

## Supplementary Materials

The following supporting information can be downloaded at: https://www.mdpi.com/article/10.3390/ph18040526/s1, Figure S1. Forest plot of mean difference in wound closure rate at 7 days after injury following RADA16 interventions in diabetic or non-diabetic skin wound models in comparison to controls (saline or without treatment); Figure S2. Forest plot of mean difference in wound closure rate at 14 days after injury following RADA16 interventions in diabetic or non-diabetic skin wound models in comparison to controls (saline or without treatment); Table S1: Primary quantitative results of the selected studies; Table S2: Eligibility criteria for the included studies; Table S3: Search strategy in the selected databases.

## Abbreviations

The following abbreviations are used in this manuscript:

PRISMA	Preferred Reporting Items for Systematic Reviews and Meta-Analyses
ROB	Risk of bias
SYRCLE	Systematic Review Centre for Laboratory animal Experimentation
MD	Mean Difference
CI	Confidence Interval
RADA16	RADARADARADARADA
3D	Three-dimensional
ECM	Extracellular matrix
PROSPERO	International Prospective Register of Systematic Reviews
PICOS	Population, intervention, comparison, outcome, and study design
MeSH	Medical subject heading
