# Supplementary material for: Efficacy of RADA16-Based Self-Assembling Peptides on Wound Healing: A Meta-Analysis of Preclinical Animal Studies
Source: Pharmaceuticals (Basel). 2025 Apr 3;18(4):526. doi: 10.3390/ph18040526 (PMC12030742; doi:10.3390/ph18040526)
Supplement: Supplementary file 1 [file pharmaceuticals-18-00526-s001.zip › Supplementary figures and tables.pdf]

Study or Subgroup	RADA16 Mean	RADA16 SD	RADA16 Total	Control Mean	Control SD	Control Total	Weight	Mean Difference IV, Random, 95% CI
**1.1.1 non-diabetic**								
Meng 2009	11.18	2.82	6	-5.24	5.72	6	32.1%	16.42 [11.32, 21.52]
Santhini 2022	55.07	2.74	9	51.23	2.47	9	35.2%	3.84 [1.43, 6.25]
**Subtotal (95% CI)**			**15**			**15**	**67.3%**	**9.92 [-2.40, 22.24]**
Heterogeneity: Tau^2 = 74.98; Chi^2 = 19.09, df = 1 ($P < 0.0001$); I^2 = 95%								
Test for overall effect: $Z = 1.58$ ($P = 0.11$)								
**1.1.2 diabetic**								
Kim 2022	46.7	1.78	6	32.49	5.58	6	32.7%	14.21 [9.52, 18.90]
**Subtotal (95% CI)**			**6**			**6**	**32.7%**	**14.21 [9.52, 18.90]**
Heterogeneity: Not applicable								
Test for overall effect: $Z = 5.94$ ($P < 0.00001$)								
**Total (95% CI)**			**21**			**21**	**100.0%**	**11.27 [2.58, 19.97]**
Heterogeneity: Tau^2 = 54.46; Chi^2 = 28.47, df = 2 ($P < 0.00001$); I^2 = 93%								
Test for overall effect: $Z = 2.54$ ($P = 0.01$)								
Test for subgroup differences: Chi^2 = 0.41, df = 1 ($P = 0.52$), I^2 = 0%								

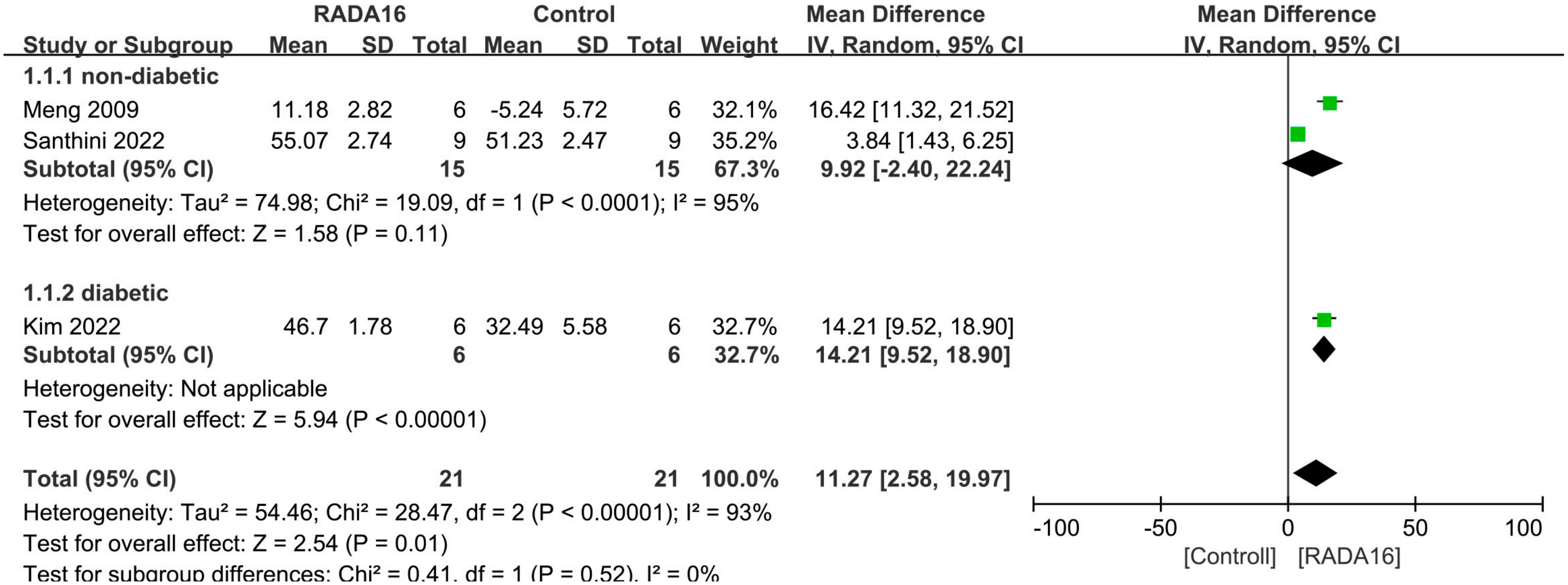

Figure S1. Forest plot of mean difference of wound closure rate at 7 days after injury following RADA16 interventions in diabetic or non-diabetic skin wound models in comparison to controls (saline or without treatment) [20,26,30].

Study or Subgroup	RADA16 Mean	RADA16 SD	RADA16 Total	Control Mean	Control SD	Control Total	Weight	Mean Difference IV, Random, 95% CI
**1.2.1 non-diabetic**								
Meng 2009	28.1	3.48	6	14.05	3.23	6	33.3%	14.05 [10.25, 17.85]
Santhini 2022	90.55	4.38	9	80.27	4.11	9	33.0%	10.28 [6.36, 14.20]
**Subtotal (95% CI)**			**15**			**15**	**66.3%**	**12.20 [8.50, 15.89]**
Heterogeneity: Tau² = 3.22; Chi² = 1.83, df = 1 (P = 0.18); I² = 45%								
Test for overall effect: Z = 6.47 (P < 0.00001)								
**1.2.2 diabetic**								
Kim 2022	68.53	1.52	6	64.47	4.31	6	33.7%	4.06 [0.40, 7.72]
**Subtotal (95% CI)**			**6**			**6**	**33.7%**	**4.06 [0.40, 7.72]**
Heterogeneity: Not applicable								
Test for overall effect: Z = 2.18 (P = 0.03)								
**Total (95% CI)**			**21**			**21**	**100.0%**	**9.44 [3.62, 15.26]**
Heterogeneity: Tau² = 22.70; Chi² = 14.13, df = 2 (P = 0.0009); I² = 86%								
Test for overall effect: Z = 3.18 (P = 0.001)								
Test for subgroup differences: Chi² = 9.42, df = 1 (P = 0.002), I² = 89.4%								

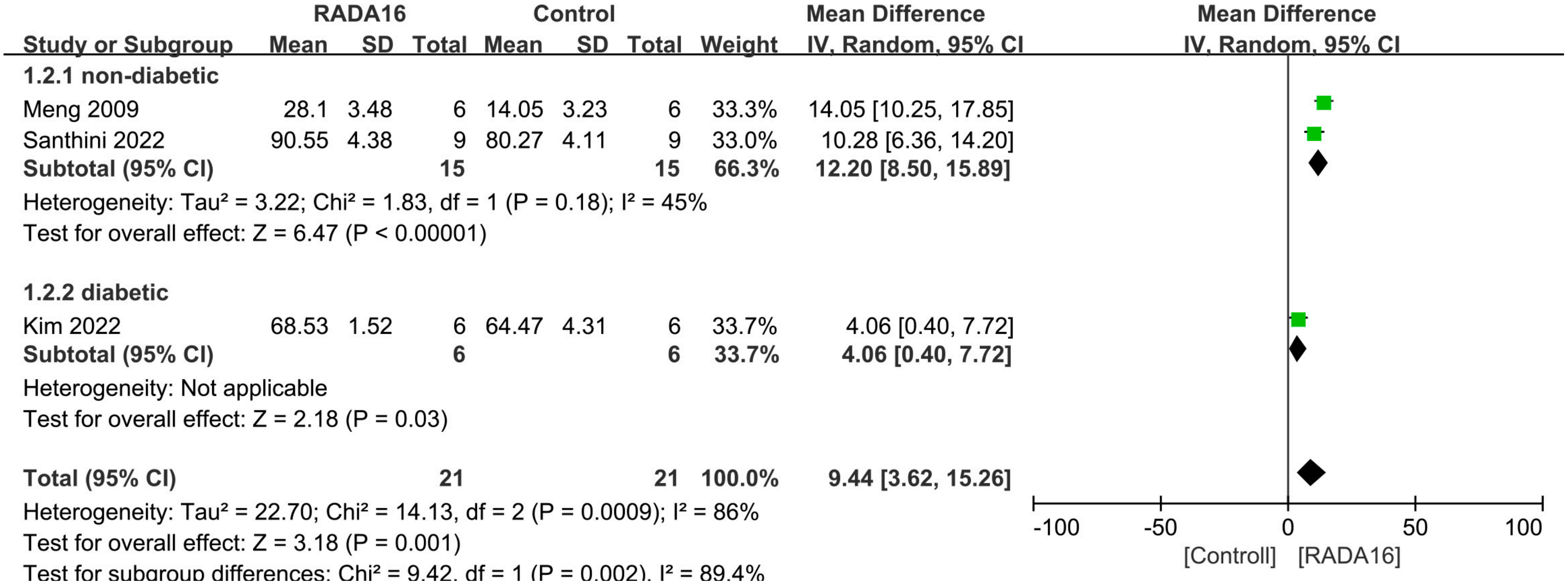

Figure S2. Forest plot of mean difference of wound closure rate at 14 days after injury following RADA16 interventions in diabetic or non-diabetic skin wound models in comparison to controls (saline or without treatment) [20,26,30].

Table S1. Primary quantitative results of the selected studies.

Study	Wound closure rate	Collagen density	Thickness of epidermis	Ki-67 expression	Hair follicle growth rate	EGF expression	FGF expression	CD31 relative expression	vWF^{+} cell density	SMA^{+} cell density	Concentration of IL-6	Concentration of IL-10	Concentration of TGF-α	Hemostasis
Deptuła [27]	√	√	√											
Dzierżyńska [28]	√	√	√											
Feng [19]	√													√
Hsu [25]														√
Meng [20]	√					√	√							
Santhini [30]	√													
Wang2020 [21]					√	√	√							
Wang2022 [22]											√		√	√
Kim2018 [29]		√		√					√	√				
Kim2022 [26]	√		√											
Xue [23]	√							√			√	√	√	
Yang [24]	√							√						

Table S2. Eligibility criteria for the included studies. PICOS schema: Population (P), Intervention (I), Comparison (C), Outcomes (O) and Study Design (S).

	**Eligibility criteria for in vivo studies**
Population	*In vivo* animal model (any species, age, and gender)
Intervention	Wound healing with application of RADA16-based self-assembling peptides as treatment
Comparison	Control, placebo, and standard treatments for skin wounds
Outcomes	Measures to assess wound healing
Study design	Preclinical animal studies

Table S3. Search strategy in the selected databases.

**Pubmed Search**		**Results**
#1	self-assembling peptide [MeSH Terms]	2,275
#2	self-assembling peptide*[Title/Abstract]	1,550
#3	RADA*[Title/Abstract]	9,731
#4	RADA16[Title/Abstract]	173
#5	SAP[Title/Abstract]	16,785
#6	PuraMatrix[Title/Abstract]	100
#7	#1 or #2 or #3 or #4 or #5 or #6	28,939
#8	wound healing [MeSH Terms]	148,004
#9	wound healing*[Title/Abstract]	98,254
#10	skin regeneration*[Title/Abstract]	1,875
#11	skin rejuvenation*[Title/Abstract]	1,086
#12	skin repair*[Title/Abstract]	914
#13	#8 or #9 or #10 or #11 or #12	207,227
#14	animal[MeSH Terms]	27,244,280
#15	animal*[Title/Abstract]	1,306,679
#16	preclinical*[Title/Abstract]	165,665
#17	in vivo*[Title/Abstract]	1,107,544
#18	#14 or #15 or #16 or #17	27,610,171
#19	#7 and #13 and #18	161
